# Derivation of Myoepithelial Progenitor Cells from Bipotent Mammary Stem/Progenitor Cells

**DOI:** 10.1371/journal.pone.0035338

**Published:** 2012-04-13

**Authors:** Xiangshan Zhao, Gautam K. Malhotra, Hamid Band, Vimla Band

**Affiliations:** 1 Department of Genetics, Cell Biology and Anatomy, University of Nebraska Medical Center, Omaha, Nebraska, United States of America; 2 Department of Microbiology and Pathology, University of Nebraska Medical Center, Omaha, Nebraska, United States of America; 3 Department of Biochemistry and Molecular Biology, University of Nebraska Medical Center, Omaha, Nebraska, United States of America; 4 Department of Pharmacology and Experimental Neuroscience, College of Medicine, University of Nebraska Medical Center, Omaha, Nebraska, United States of America; 5 The Eppley Institute for Research in Cancer and Allied Diseases, University of Nebraska Medical Center, Omaha, Nebraska, United States of America; Wayne State University School of Medicine, United States of America

## Abstract

There is increasing evidence that breast and other cancers originate from and are maintained by a small fraction of stem/progenitor cells with self-renewal properties. Recent molecular profiling has identified six major subtypes of breast cancer: basal-like, ErbB2-overexpressing, normal breast epithelial-like, luminal A and B, and claudin-low subtypes. To help understand the relationship among mammary stem/progenitor cells and breast cancer subtypes, we have recently derived distinct hTERT-immortalized human mammary stem/progenitor cell lines: a K5^+^/K19^−^ type, and a K5^+^/K19^+^ type. Under specific culture conditions, bipotent K5^+^/K19^−^ stem/progenitor cells differentiated into stable clonal populations that were K5^−^/K19^−^ and exhibit self-renewal and unipotent myoepithelial differentiation potential in contrast to the parental K5^+^/K19^−^ cells which are bipotent. These K5^−^/K19^−^ cells function as myoepithelial progenitor cells and constitutively express markers of an epithelial to mesenchymal transition (EMT) and show high invasive and migratory abilities. In addition, these cells express a microarray signature of claudin-low breast cancers. The EMT characteristics of an un-transformed unipotent mammary myoepithelial progenitor cells together with claudin-low signature suggests that the claudin-low breast cancer subtype may arise from myoepithelial lineage committed progenitors. Availability of immortal MPCs should allow a more definitive analysis of their potential to give rise to claudin-low breast cancer subtype and facilitate biological and molecular/biochemical studies of this disease.

## Introduction

The epithelial compartment of the mammary gland is composed of two types of cells, luminal cells that line the ductal tree and form the secretory epithelial cells within the alveoli of a lactating mammary gland, and outer myoepithelial cells that border the basal lamina separating epithelial cells from the extracellular matrix. While differentiated myoepithelial cells resemble smooth muscle cells, they exhibit markers of epithelial cells, such as cytokeratins [1]–[3]. The relationship of luminal epithelial cells with breast cancer has received considerable attention as tumor cells in most human breast cancers share features of luminal cells. In contrast, the relationship of myoepithelial cells with oncogenesis is less clear. Certain findings suggest that myoepithelial cells play a role in suppressing mammary oncogenesis: i) myoepithelial cells have been shown to secrete a number of suppressor proteins that limit cancer cell growth and invasiveness [4], [5]; ii) compared to the frequency of human breast cancers that share features of luminal cells, neoplasms of apparent myoepithelial origin, such as myoepithelioma [6] or metaplastic tumors [7], are extremely rare.

Breast cancer is a clinically heterogeneous disease [8], [9]. Previous expression profiling studies have further expanded the concept of clinical heterogeneity and identified five major subtypes of breast cancer: basal epithelial-like, ErbB2-overexpressing, normal breast epithelial-like and two luminal (luminal A and B) subtypes [9]–[11]. Notably, analyses of patient survival have shown significantly different outcomes for patients belonging to various subtypes [9], [11].

It is unclear whether distinct cells of origin contribute to the heterogeneity of breast cancer and which cell types are most susceptible to oncogenesis [12]. The correspondence of some breast cancer subtypes with cell types present in the normal mammary gland (such as luminal) strongly supports the idea that breast cancer subtypes may represent malignancies of biologically distinct cell types. Alternatively, different subtypes of breast cancers may arise from a common precursor based on distinct pathways of oncogene-driven reprogramming [12].

Heterogeneity of breast cancers is closely linked to tumor progression, metastasis and treatment failure, traits traditionally ascribed to clonal evolution as a result of inherent genomic instability of tumor cells and tumor-host interactions [13]. The stem cell hypothesis however suggests an alternate explanation with tumor heterogeneity reflecting the relative fraction of cancer stem/progenitor cells and differences in their abilities to produce progeny at various stages of differentiation [14].

Recent molecular analyses have added further heterogeneity to breast cancer by identifying a new, claudin-low subtype with poor prognosis comparable to that associated with the basal subtype [15]–[17]. However, the origin of claudin-low breast cancers remains unclear. Here, we present evidence that myoepithelial lineage restricted K5^−^/K19^−^ myoepithelial progenitor cells (MPCs) derived from bipotent K5^+^/K19^−^ stem/progenitor cells share a molecular gene expression signature with claudin-low breast cancer subtype. Furthermore, MPCs express markers of epithelial to mesenchymal transition (EMT) and exhibit higher capacity to migrate and invade compared to more primitive precursors. Our analyses suggest that claudin-low breast cancer subtype may originate from or acquire characteristics of MPCs that exhibit EMT as an intrinsic property. The immortal MPCs generated in this study may also be useful future cellular tools to further characterize the biology of claudin-low breast cancer subtype upon inducing oncogenesis.

## Materials and Methods

### Cell culture

The hTERT-immortalized K5^+^/K19^−^ and MPCs were grown in the DFCI-1 (D) medium, as described [18], [19].

### Antibodies

Mouse anti-claudin-1 (sc-81796) monoclonal, anti-human K19 (sc-6278), K8 (sc-8020), α–smooth muscle actin (sc-3225), Twist (81417), GATA-3 (sc-268) and vimentin (sc-6260) antibodies were purchased from Santa Cruz Biotechnology. Rabbit anti-claudin-3 (34–1700) or mouse anti-claudin-4 (329400) monoclonal antibodies were purchased from Invitrogen. Mouse anti-occludin (611091), mouse anti-fibronectin (610077), MUC1 (550486), and mouse CD29 (61047) were purchased from BD Bioscience. CD49f (CBL458), was from Chemicon International; CD90 (Thy-1) (MS-1013-p) from Lab Vision; and ER (VP-E613) from Vector Laboratories. Rabbit anti-human K5 (RB-160P) was from Covance, mouse anti-human CD10 (NCL-L-CD10-270), K5 (NCL-L-CK5), K14 (NCL-L-LL02), and K18 (NCL-C51) were from Novocastra Laboratories. P63 ab-1(4A4), mouse, MS-1081-p were from Neo- Marker; rabbit anti-human vimentin (clone sp20, RM-9120-S0) from Thermo Scientific; and mouse anti-human β-actin (AC-15) was purchased from Abcam.

### Isolation of MPC cells

As described earlier, serially clonally derived K5^+^/K19^−^ cells [19] were seeded at low density (300 cells/100-mm dish) in a 3D Matrigel cultures (BD Bioscience), as described previously [20]. Cells were allowed to grow for 10 days and supplemented with fresh medium containing 2% matrigel every two days. In this culture system, single cells form clonal acinus structures. Single acini were then isolated, trypsinized, and gradually expanded from 96-well to 24-well plates and finally to T-25 flask. Morphologically distinct colonies were isolated and characterized for various markers using western blotting to identify K5^−^/K19^−^ clones, as described in results section.

### Affymetrix Chip-Based Microarray Analyses

Total RNA was isolated using the Trizole reagent. A total of 200 ng of total RNA from a representative MPC clone was reverse transcribed and cRNA generated per manufacturer's instructions using the Affymetrix 3′ IVT Express labeling kit (Affymetrix). Resultant cRNA probes were hybridized to the Affymetrix human U133Plus 2 genome array per manufacture's suggestions and the chips were scanned using a Gene Chip 3,000 6G scanner through UNMC DNA Microarray Core Facility. The resultant data sets were scaled using GCOS software, evaluated with respect to quality assurance parameters to include background, hybridization kinetics, and reverse transcription efficiency. The complete microarray data of MPCs is submitted to Gene Expression Omnibus (GEO) database accession number GSE34440. The parental bipotent K5^+^/K19^−^ cell microarray data (accession number GSE22580) was described previously [19].

### Western blot analysis

Cell lysates were prepared using 1× SDS sample buffer, quantified using the BCA protein assay kit (Pierce) and subjected to western blotting using the indicated antibodies, as described above.

### Immunofluorescence staining

Cells were grown on uncoated coverslips (for cells plated in MEGM medium (differentiation media) or DFCI-1 medium), fixed in 4% paraformaldehyde, permeabilized with 0.3% Triton X-100 and blocked in 5% donkey serum. The coverslips were then incubated with primary antibodies for 2–3 h followed by Alexa Flour 594-conjugated donkey anti-mouse (1∶1,000) antibody for 1 h. The slides were mounted and images obtained under a fluorescence microscope (Zeiss Axioplan 2 imaging microscope, 20× objective).

### Migration assay

The cells were trypsinized, resuspended in growth factor deprived DFCI-3 (D3) medium [18], and 5×10^4^ cells/well were added to the top of transwell chambers with an 8 µm pore size filter (BioCoat chambers; BD Biosciences). After 10 min, DFCI-1 medium was added to the lower chamber and incubated for 13 h. The cells on top of the membrane (not migrated) were removed and the migrated cells at the bottom surface of the membrane were visualized by staining with Diff-Quik Stain Set kit (Siemens Healthcare Diagnostics Inc.) and counted using an inverted tissue culture microscope.

### Invasion assay

The invasion assay was done as above for the migration assay except that cells were seeded on top of Matrigel-coated chambers (BD invasion chambers; 8 µm pore size filter; BD Biosciences). The cells were incubated for 15 h prior to counting cells at the bottom surface as above.

### Anchorage-independent colony formation assay

2 ml of 0.7% agarose in growth medium was allowed to solidify as the bottom layer in wells of a six-well plate, and 10^5^ cells in 2 ml of 0.3% agarose in growth medium were added as the top layer. The images were obtained 30 days after cell seeding.

### Three-dimensional (3D) Matrigel Cultures

2000 cells were mixed with 200 µl matrigel and added to a 60 µl matrigel coated well of a 24 well plate. The cells were cultured with DFCI-1 medium. Images were acquired under a Nikon inverted microscope after 12 days culture.

## Results

### Derivation of K5^−^/K19^−^ cells with myoepithelial markers from K5^+^/K19^−^ stem/progenitor mammary epithelial cells

**Figure 1:**
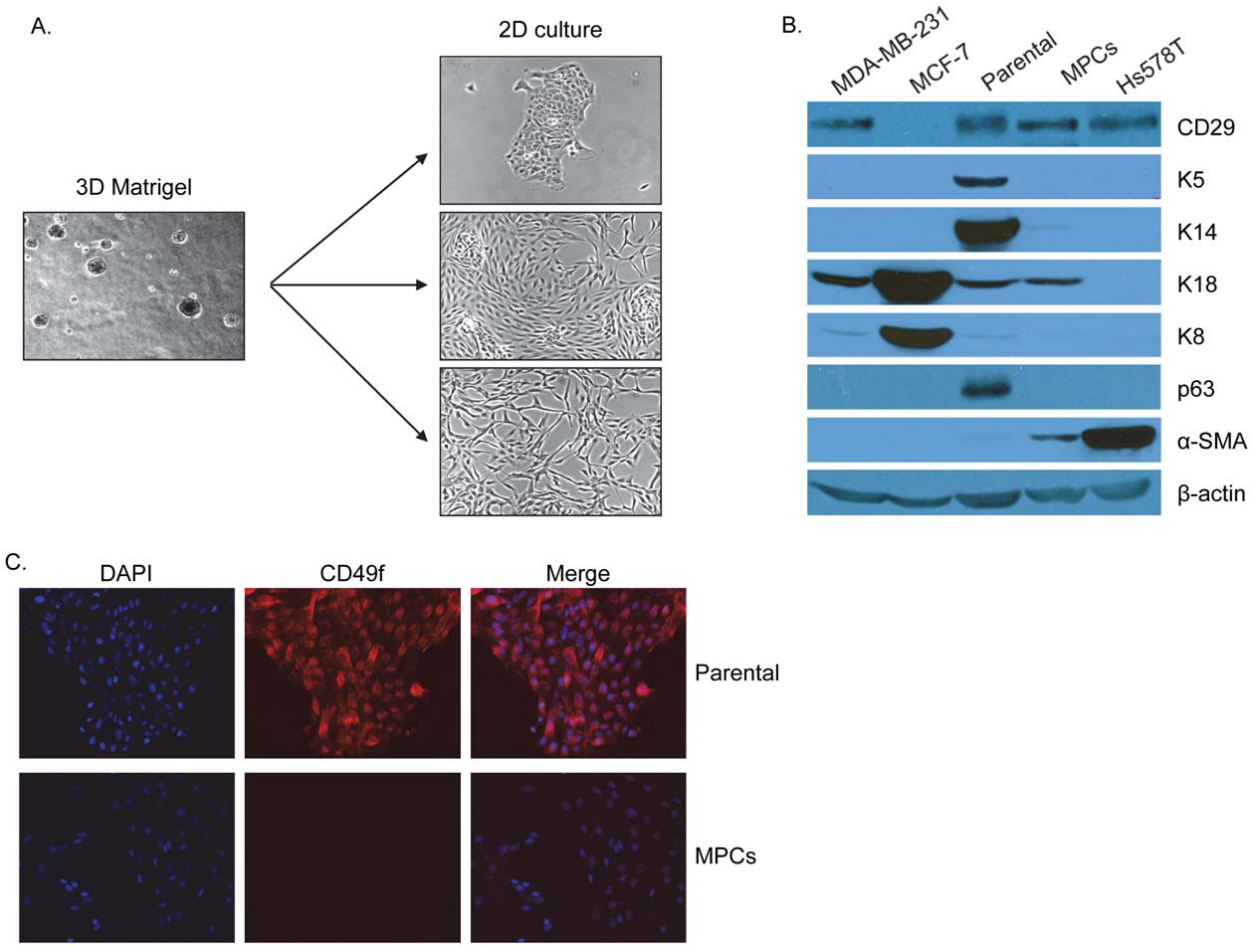
Isolation of MPCs (K5^−^/K19^−^) cells from bipotent parental (K5^+^/K19^−^) cells and stem/progenitor cell markers analysis. (**A**) Bipotent parental cells were seeded at low density in a 3D Matrigel cultures. Shown here are phase contrast morphologies of three colonies from 3D cultures, when transferred to 2D cultures. (**B**) Western blotting of parental and MPCs using indicated antibodies. Breast cancer cell lines MDA-MB-231, MCF-7, and Hs578T cells are used as controls. (**C**) Immunofluorescence staining of parental and MPCs using CD49f antibody (red), blue nuclei are stained with DAPI.

We have recently described two types (K5^+^/K19^−^ or K5^+^/K19^+^) of hTERT-immortalized human mammary stem/progenitor cell lines, both of which exhibit marker profiles of bipotent mammary stem/progenitor cells, and demonstrate the abilities of self-renewal as well as differentiation into luminal and myoepithelial cells when cultured under appropriate culture conditions [19]. We and others have also shown that mammary stem/progenitor cells exhibit self-renewal and differentiation abilities when grown in 3D Matrigel cultures [21]–[24]. We therefore seeded K5^+^/K19^−^ bipotent progenitor cells at low density in DFCI-1 medium in Matrigel culture, manually picked individual colonies and propagated these in regular 2D culture in DFCI-1 medium [18], [25]. We observed either tight colonies similar to the self-renewing assemblies of parental cells, colonies in which all cells exhibited a spindle-shaped morphology, or colonies with a mixture of both tight epithelial cells and spindle-shaped cells (Fig. 1A). These spindle shaped colonies are reminiscent of peripheral cells that exhibit myoepithelial characteristics when parental cells are cultured in the MEGM medium to induce differentiation, as we have previously shown [19]. However, unlike differentiated myoepithelial cells that appear in MEGM medium [19], the clonal lines with spindle-shaped morphology continued to proliferate in culture and could be passaged indefinitely. It is important to mention that isolation of K5^−^/K19^−^ cells is not the result of a heterogeneous population already present in parental K5^+^/K19^−^ cells as published previously, we had serially cloned K5^+^/K19^−^ cells from hTERT-immortalized hMECs [19].

To discern the relationship of the morphologically distinct (spindle-shaped) cell population, we characterized these for the expression of lineage and differentiation-related markers as compared to their parental cells, using western blotting. Compared to parental mammary stem/progenitor line, the spindle-shaped cells showed a loss or dramatically reduced expression of keratin (K) 5, K14, p63 and CD49f, indicating that these cells were phenotypically distinct from the parental K5^+^/K19^−^ cell line (Fig. 1B and C). Like the parental cells, the spindle shaped cells isolated are also K19^−^ (data not shown). Notably, the spindle shaped cells continued to express CD29 (Fig. 1B) and CD44 (CD44 data not shown), both well-known mammary stem cell markers, and maintained the weak expression of luminal markers K8 and K18. However, compared to undetectable levels of alpha smooth muscle actin (α-SMA, a known myoepithelial marker) in the parental cell line, the spindle-shaped cells showed readily detectable α-SMA signals (Fig. 1B). Co-expression of stem cell (CD29 and CD44), luminal (K8, K18) and myoepithelial (α-SMA) markers suggests that the spindle-shaped progeny of the parental K5^+^/K19^−^ line represent a progenitor population. We designated this population of cells K5^−^/K19^−^ to distinguish them from the K5^+^/K19^−^ or K5^+^/K19^+^ bipotent stem/progenitor cells we have previously identified and published [19]. Significantly, in multiple experiments we could reproducibly derive the spindle-shaped K5^−^/K19^−^ population from K5^+^/K19^−^ parental lines, whereas we could not isolate such cells from K5^+^/K19^+^ lines, even though both types of stem/progenitors are capable of differentiating into luminal as well as myoepithelial lineages when cultured in MEGM medium [19].

### K5^−^/K19^−^ cells indefinitely maintain self-renewal and exhibit unipotent myoepithelial differentiation upon induction

**Figure 2:**
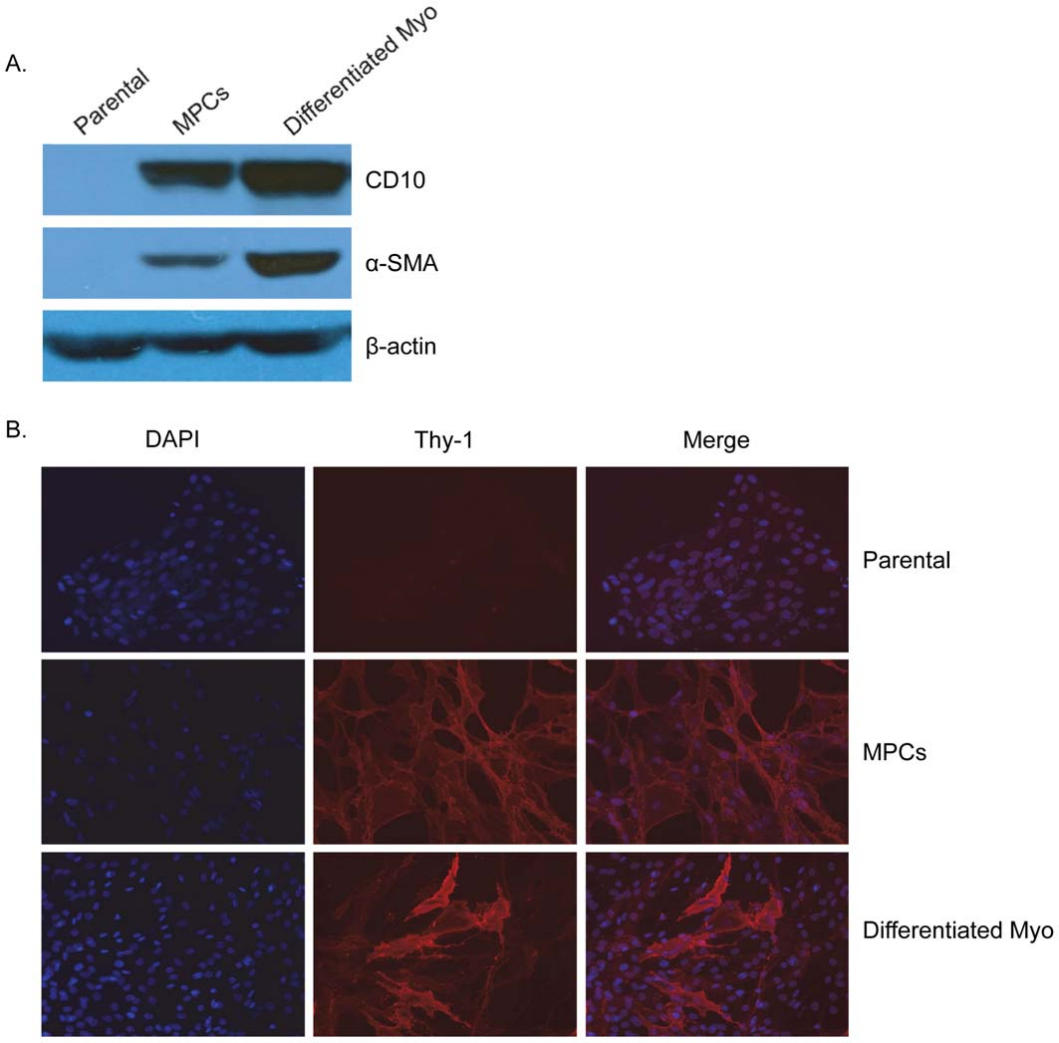
Comparison of myoepithelial markers in MPCs and terminally differentiated myoepithelial cells. (**A**) Western blotting of lysates prepared from bipotent parental cells, MPC, and differentiated myoepithelial cells, using antibodies against the myoepithelial markers CD10 and α-SMA. (**B**) Immunofluorescence staining of bipotent parental cells, MPC, and differentiated myoepithelial cells using the myoepithelial marker Thy-1 (red), blue nuclei are stained with DAPI.

To test the bipotent differentiation potential of K5^−^/K19^−^ hMEC population suggested by their stable co-expression of stem, luminal and myoepithelial cell markers we cultured these in the MEGM differentiation medium. Under these conditions, the spindle-shaped cells continued to grow, suggesting their ability to self-renew. Western blot comparison of parental, K5^−^/K19^−^ and differentiated myoepithelial cells (derived from K5^−^/K19^−^) showed a marked up-regulation of the myoepithelial cell markers CD10 and α-SMA in differentiated myoepithelial cells (Fig. 2A). Immunofluorescence analyses of Thy-1, another myoepithelial marker showed an increase in the intensity of staining; the increase was seen in a variable proportion of cells, apparently reflecting more advanced myoepithelial differentiation of some cells (Fig. 2B). As expected, the bipotent parental cells did not show expression for myoepithelial differentiation markers (Fig. 2A and B). In contrast to the readily detectable myoepithelial differentiation, when K5^−^/K19^−^ cells were plated in differentiation medium, we found no evidence of luminal differentiation in repeated experiments. Based on the apparent unipotent differentiation ability of K5^−^/K19^−^ cells to undergo further transition towards full myoepithelial differentiation and their failure to undergo luminal differentiation, we suggest that these cells represent myoepithelial progenitor cells (MPCs).

### MPCs exhibit epithelial to mesenchymal transition (EMT)

**Figure 3:**
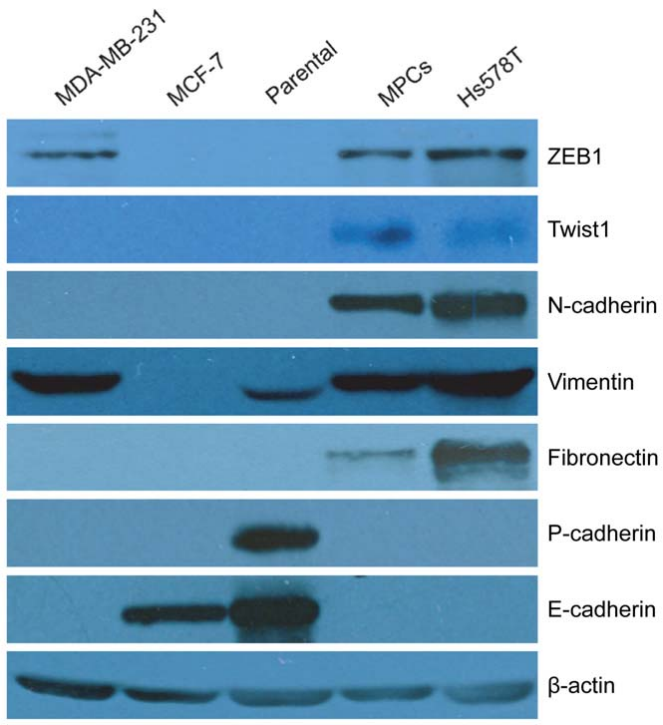
Western blotting of MPCs using EMT markers. Cell lysates from exponentially proliferating bipotent parental and MPCs were analyzed for expression of indicated EMT markers using specific antibodies. Breast cancer cell lines MDA-MB-231, MCF-7, and Hs578T were used as controls.

Based on the spindle shaped morphology of MPCs, we hypothesized that they may possess characteristics of EMT cells. To test this idea, we used western blotting to assess the expression of known EMT markers. In contrast to the parental bipotent cells, the MPCs showed loss of expression of epithelial makers E-cadherin and P-cadherin and dramatic increase in the level of vimentin expression together with de novo high level expression of ZEB1, Twist1, N-cadherin and fibronectin, all markers of mesenchymal cells (Fig. 3). As controls, a non-invasive luminal breast cancer cell line MCF-7 express E-cadherin but no mesenchymal markers, whereas MDA-MB-231 and Hs578T two known invasive breast cancer cell lines lack E- and P-Cadherin expression but express some of the mesenchymal markers tested (Fig. 3). These results clearly indicate that MPCs express EMT markers compared to their parental cells.

### MPCs express a molecular signature of claudin-low subtype of breast cancer

**Figure 4:**
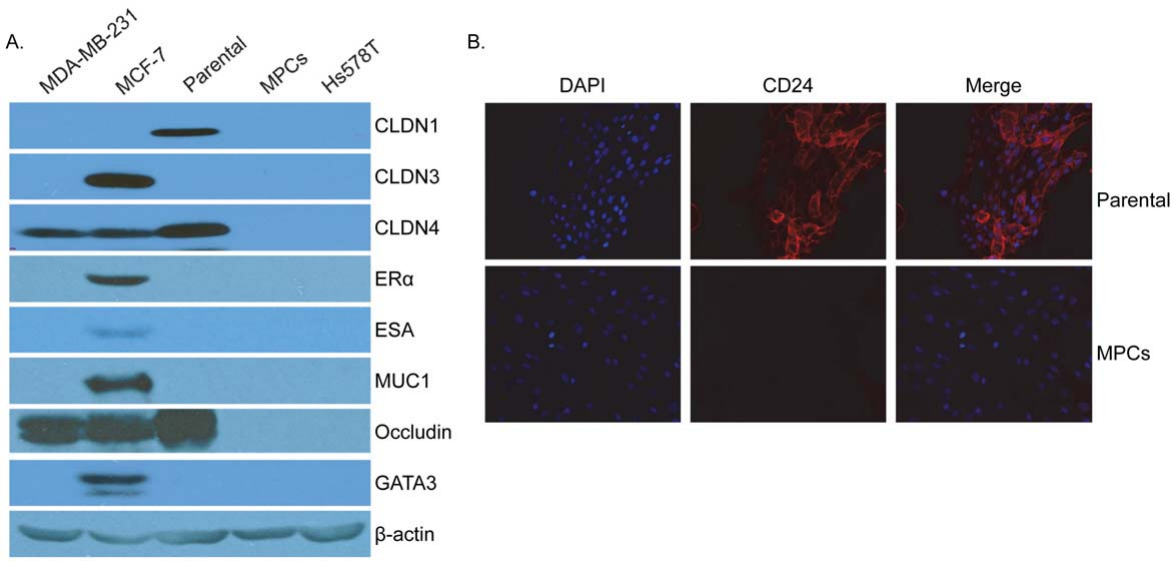
Western blot and immunofluorescence analysis of parental bipotent cells and MPCs using various markers from the signature of claudin-low breast cancer subtype. (**A**) Western blot analysis of cell lysates from exponentially proliferating bipotent cells and MPCs were analyzed for expression of indicated markers using specific antibodies. Breast cancer cell lines MDA-MB-231, MCF-7, and Hs578T were used as controls. (**B**) Immunofluorescence staining using CD24 antibody (red), nuclei (blue) represent DAPI staining.

Evidence presented above demonstrates that MPCs are distinct from other stem/progenitor cell types. To further characterize their distinctive features, we performed microarray analysis of MPCs with their parental bipotent cell type. Using the published claudin-low gene expression signature [15], [17] we observed that MPCs exhibit the same differential gene expression patterns as the claudin-low subtype of breast cancer (Table 1). We used western blot and immunofluorescence analyses to verify the expression of a number of genes associated with the claudin-low signature. These analyses confirmed the loss of expression of claudins 1 and 4, and occludin in MPC as compared to parental bipotent cells (Fig. 4A). In addition, both parental bipotent and MPCs do not express proteins such as, claudin 3, ER, ESA, MUC1 and GATA3 (Fig. 4A), that are known signature of claudin-low breast cancers. Furthermore, as shown above MPCs lack expression of CD24 (Fig. 4B), K5, K14 (Fig. 1B), CD49f (Fig. 1C) and E-cadherin (Fig. 3), further reinforcing their signature of claudin-low breast cancers. Taken together the expression profile mentioned above, and an increase in expression of CD10 (Fig. 2A), Thy-1 (Fig. 2B), ZEB1, Twist1 and Vimentin (Fig. 3), underscores that MPCs share a molecular signature with claudin-low subtype of breast cancer. Taken together, these results demonstrate that MPCs have a signature of the claudin-low subtype of breast cancers.

**Table 1:** Comparison of claudin-low gene signature from microarray analysis of parental bipotent cells and MPCs.

Gene Symbol	Gene Title	Parental mas5_Signal	MPCs mas5_Signal
CLDN1	claudin 1	500.5	1.9
CLDN3	claudin 3	20.1	20.4
CLDN4	claudin 4	616.9	29.6
CLDN7	claudin 7	881	45.6
CDH1	cadherin 1, type 1, E-cadherin (epithelial)	19103.8	2.5
OCLN	occludin	354.6	49.9
VIM	vimentin	15990.8	28292.7
ZEB1	zinc finger E-box binding homeobox 1	43.2	5205.2
ZEB2	zinc finger E-box binding homeobox 2	20	528.2
TWIST1	twist homolog 1 (Drosophila)	357.8	9140.2
TWIST2	twist homolog 2 (Drosophila)	714.9	2617.4
KRT5	keratin 5	18257.2	499.1
KRT14	keratin 14	49967.4	2021.5
KRT17	keratin 17	29653	936.2
KRT18	keratin 18	8268.7	5619.3
KRT19	keratin 19	4.4	1.3
ESR1	estrogen receptor 1	12	55.3
PGR	progesterone receptor	8.5	4.9
GATA3	GATA binding protein 3	277	13.6
ERBB2	epidermal growth factor receptor 2	44.6	48.4
CD44	CD44 molecule (Indian blood group)	6799.4	2800.3
CD24	CD24 molecule	5808.7	10.7
MME(CD10)	membrane metallo-endopeptidase	966.7	8419.2
ITGA6(CD49f)	integrin, alpha 6	3766	329.9
ITGB1(CD29)	integrin, beta 1	32483.1	29343.6
MUC1	mucin 1, cell surface associated	135.8	143.9
THY1	Thy-1 cell surface antigen	8.4	1403.8

Shown here are MAS5 normalized expression signals of claudin-low signature genes from our microarray analysis upon differentiation of bipotent parental (K5^+^/K19^−^) cells to unipotent MPCs (K5^−^/K19^−^).

### MPCs exhibit higher migration and invasion

**Figure 5:**
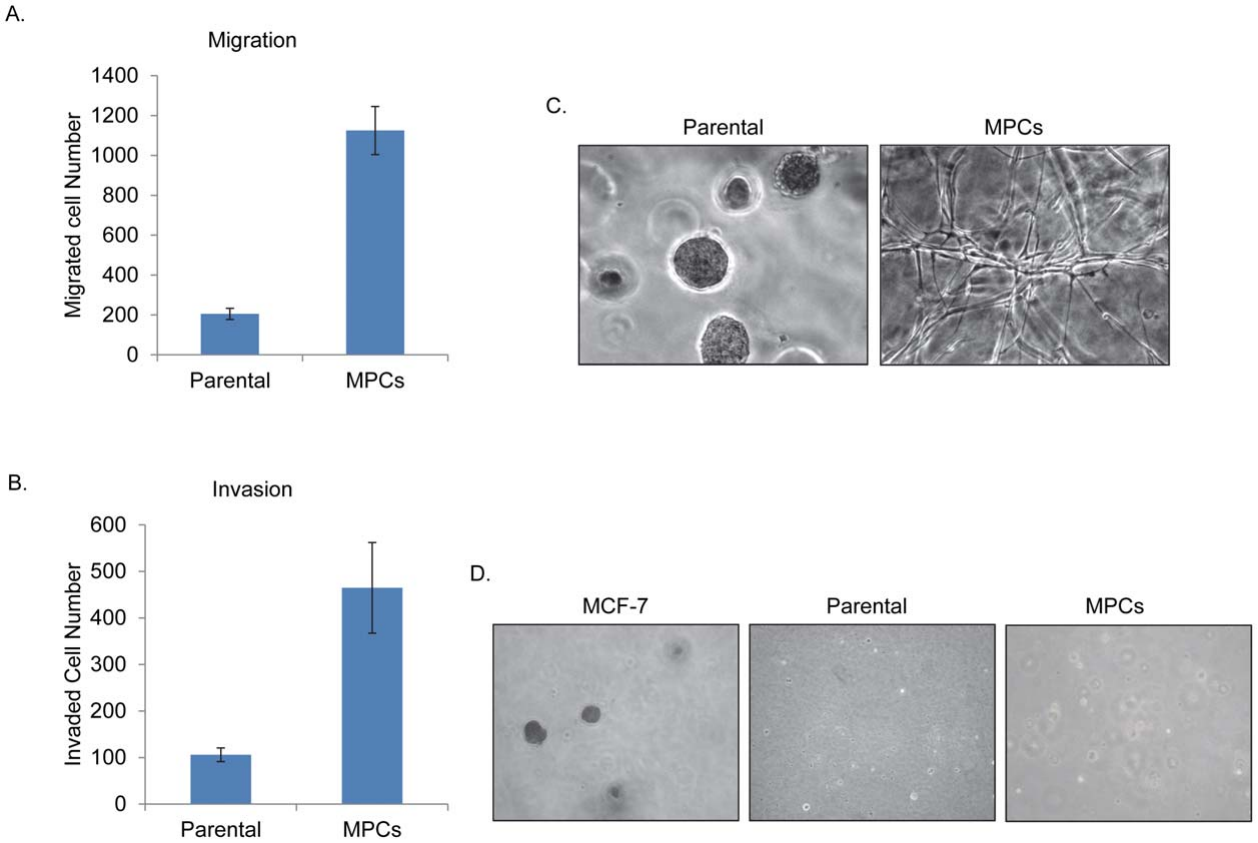
Comparison of parental bipotent cells and MPCs for invasion, migration, anchorage independence and 3D proliferation in matrigel. (**A**) Migration assay using transwell chambers were performed on exponentially proliferating indicated cells. Shown here is a bar diagram of the number of cells that migrated in both cell types. The data represents the mean^+^/_−_ standard deviation (SD) of three experiments done in six replicates. (**B**) Invasion assay were performed with indicated cells using matrigel coated transwell chambers. Shown here is a bar diagram of number of cells that invaded in both cell types. The data represent mean^+^/_−_ SD of three experiments done in six replicates. (**C**) 3D matrigel culture. Shown here are phase contrast pictures at day12 using an inverted microscope. (**D**) Exponentially proliferating cells were analyzed for anchorage independence by soft agar assay. Shown here are images of colonies after 30 days, using inverted microscope.

Given the expression of markers of EMT in MPCs, and the known association of EMT with increased migration/invasion abilities of cells [26], [27], we compared the parental bipotent cells with MPCs for migration and invasion using transwell chambers. Indeed, the MPCs exhibit substantially elevated levels of cell migration (Fig. 5A) and invasion through Matrigel (Fig. 5B) when compared with parental bipotent cells.

### MPCs form branching structures instead of acini or ductal structures in 3D cultures

As we and others have shown, the mammary stem/progenitor cells form acinar structure in 3D Matrigel cultures, consistent with establishment of polarized epithelial cell layers [21], [24]. EMT is associated with a loss of polarity and loss of acinar/ductal formation on Matrigel [28]. We therefore compared the parental bipotent cells and the MPCs ability to form structures in 3D Matrigel culture. Notably, while the parental cells expectedly formed acinar structures, MPCs formed branching structures (Fig. 5C) consistent with their increased migratory and invasive properties. While the MPCs exhibit EMT and loss of polarized acinar growth in 3D culture, neither their parental cells nor the MPCs exhibit anchorage-independent growth, a characteristic of oncogenically transformed cells, when cultured on soft agar (Fig. 5D).

## Discussion

Luminal and myoepithelial components of the mammary gland share their developmental origin yet very little is known about molecular pathways involved in the generation of myoepithelial cells and their precise relation to human breast cancer remains relatively unclear and under-explored. Here, we describe the isolation of immortal human mammary progenitor cells that stably express characteristics consistent with myoepithelial progenitor cells (MPCs). Molecular and functional characterization of the MPCs we established here demonstrate that they exhibit EMT characteristics and molecular signatures shared with claudin-low breast cancers raising the possibility that claudin-low breast cancer subtype arises from myoepithelial progenitors.

Previously we established human mammary epithelial stem/progenitor cell lines with K5^+^/K19^+^ or K5^+^/K19^−^ phenotype both of which exhibit self-renewal potential and the ability to differentiate into luminal as well as myoepithelial progeny [19]. In this study, we demonstrate that K5^+^/K19^−^ cell type differentiate to K5^−^/K19^−^ cells that exhibit myoepithelial lineage characteristics. Co-expression of luminal, myoepithelial and stem cell markers strongly suggested that these cells represented progenitor cells rather than terminally-differentiated myoepithelial cells. As MPC were derived from clonal bipotent parental cells, this eliminated the possibility of heterogeneity within the parental population. While parental cells yield both luminal and myoepithelial progeny when cultured in MEGM differentiation medium, K5^−^/K19^−^cells only yielded further differentiation along the myoepithelial lineage with upregulation of myoepithelial markers. Thus, we conclude that stable K5^−^/K19^−^ progeny from the K5^+^/K19^−^ mammary stem/progenitors represents myoepithelial progenitor cells (MPCs).

The derivation of MPC from bipotent K5^+^/K19^−^ cells, but our inability to obtain such cells from bipotent K5^+^/K19^+^ cells suggest that we may not have yet identified the appropriate *in vitro* conditions for generating MPCs from K5^+^/K19^+^cells in matrigel or that K5^+^/K19^+^cells lack the ability to differentiate into MPCs under these conditions. This important distinction for lineage relationships and committed states of human mammary stem and progenitor cells will require detailed future studies.

We observed that MPCs exhibit markers of EMT and showed elevated levels of cell migration and invasion compared to their parental cells. The linkage of EMT in mammary epithelial cells with elevated cell migration and invasion has been examined primarily in the context of cancer cells due to the potential importance of these traits in tumor metastasis [26], [27] and radio- and chemo-resistance [29]. However, recent studies have begun to link EMT to mammary epithelial stem cells [30]. In fact, ectopic expression of genes that promote EMT in mammary epithelial cells without EMT has been shown to promote traits associated with mammary stem cells [30]. Given our findings that two distinct types of human mammary stem/progenitors with bipotent differentiation capabilities (K5^+^/K19^−^ and K5^+^/K19^+^) do not exhibit molecular or phenotypic evidence of EMT but a unipotent MPC derived from one of these (K5^+^/K19^−^) exhibits this trait, the linkage of EMT with mammary stem cell behavior should be interpreted with caution. It is possible that EMT is either a transitional feature of mammary stem cells or a feature of certain committed progenitors, such as MPCs, as we show here. Consistent with this idea, a recent study showed that the EpCAM^pos^/CD49f^high^ subpopulation of cells within non-tumorigenic basal mammary epithelial cell lines (MCF10A and MCF12A) spontaneously attained mesenchymal-like features through EMT and do not exhibit stem cell properties [31].

Importantly, recent studies have shown that EMT is a feature of the claudin-low subtypes of breast cancers which is associated with poor prognosis and resistance to therapy [15]. Our findings that MPCs exhibit an EMT phenotype, and share a molecular signature with claudin-low breast cancers suggests the possibility that the claudin-low subtype may originate from MPCs or from a rewiring of differentiated mammary epithelial cells to a MPC-like state. In support of this theory, a recent report has shown that deliberate transformation of basal/myoepithelial cells with SV40 and K-ras results in metaplastic carcinomas resembling claudin-low tumors [7]. Regardless of the mechanisms, the potential linkage of claudin-low breast cancers with MPCs should help rethink the role of myoepithelial lineage in breast cancer. While myoepithelial cells have been suggested to protect mammary tumorigenesis [4], [5], [32], they are known to contribute to the synthesis and remodeling of the basal lamina and the basement membrane, and are known to exert paracrine effects on secretary epithelial cells [32]. Consistent with these functions, several lines of evidence suggest that myoepithelial cells regulate the progression of ductal carcinoma in situ (DCIS) to invasive breast cancer [32]. Notably, myoepithelial tumors such as myoepithelioma [33] and metaplastic carcinomas [7] are rare but aggressive. These studies underscore the importance of further research in understanding the origin and contribution of myoepithelial cells to breast cancers.

In conclusion, we have isolated a MPC population from K5^+^/K19^−^ bipotent stem/progenitor cells that exhibits unipotent myoepithelial lineage-specific differentiation. Importantly, these cells exhibit intrinsic EMT characteristics and elevated cell migration and invasion. Significantly, MPCs share a molecular signature with claudin-low breast cancers. Together, these findings suggest that MPC with EMT characteristics may represent a precursor cell type for claudin-low breast cancer.
